# Digital rectal examination skills: first training experiences, the motives and attitudes of standardized patients

**DOI:** 10.1186/s12909-015-0292-7

**Published:** 2015-02-01

**Authors:** Christoph Nikendei, Katja Diefenbacher, Nadja Köhl-Hackert, Heike Lauber, Julia Huber, Anne Herrmann-Werner, Wolfgang Herzog, Jobst-Hendrik Schultz, Jana Jünger, Markus Krautter

**Affiliations:** 1 Department of General Internal Medicine and Psychosomatics, University of Heidelberg, Medical Centre, Heidelberg, Germany; 2 Department of General Practice and Health Services Research, University Hospital Heidelberg, Heidelberg, Germany; 3 Department of Internal Medicine VI, Psychosomatic Medicine and Psychotherapy, University Hospital Tübingen, Tübingen, Germany; 4 Department of Nephrology, University of Heidelberg, INF 162, 69115 Heidelberg, Germany

**Keywords:** Intimate physical examinations, Digital rectal examination, Standardized patients, Qualitative research

## Abstract

**Background:**

Physical clinical examination is a core clinical competence of medical doctors. In this regard, digital rectal examination (DRE) plays a central role in the detection of abnormalities of the anus and rectum. However, studies in undergraduate medical students as well as newly graduated doctors show that they are insufficiently prepared for performing DRE. Training units with Standardized Patients (SP) represent one method to deliver DRE skills. As yet, however, it is little known about SPs’ attitudes.

**Methods:**

This is a qualitative study using a grounded theory approach. Interviews were conducted with 4 standardized patients about their experiences before, during and after structured SP training to deliver DRE competencies to medical students. The resulting data were subjected to thematic content analysis.

**Results:**

Results show that SPs do not have any predominant motives for DRE program participation. They participate in the SP training sessions with relatively little prejudice and do not anticipate feeling highly vulnerable within teaching sessions with undergraduate medical students.

**Conclusions:**

The current study examined SPs’ motives, views, expectations and experiences regarding a DRE program during their first SP training experiences. The results enabled us to derive distinct action guidelines for the recruitment, informing and briefing of SPs who are willing to participate in a DRE program.

## Background

The physical examination of patients constitutes a core competence of medical care, and along with the history-taking, takes place at the beginning of any patient-doctor contact. Together, the history-taking and the physical examination form the basis for a valid diagnosis, for the instigation of further necessary diagnostic steps, and for preparing a therapeutic treatment plan. A good physical examination is therefore essential for the high-quality treatment of patients [1]. At the same time, the early detection of abnormalities in the anus, the rectum and the prostate is highly relevant for further diagnosis and consequently for effective treatment. The central importance attributed to the physical examination in this respect is highlighted by the fact that abnormalities of the prostate discovered within a DRE have a positive predictive value for the presence of a prostate carcinoma of up to 30% [2]. Moreover, in around a third of cases, rectal carcinomas are palpable in a digital-rectal examination (DRE) [3].

Despite this clinical evidence, studies in final year students as well as newly graduated doctors revealed that they are insufficiently prepared for performing DRE [4-9], and if they nevertheless do perform DRE in a clinical setting, doctors are not sufficiently supervised by senior physicians [8,10]. Furthermore, final year students complain of insufficient supervision as the most relevant hindrance for the acquisition of DRE skills [7]. This seems surprising given that there are a variety of methodological teaching approaches to deliver DRE skills, such as training on part-task trainers [11], finger movement simulators including video feedback [12,13], rectal and urological teaching associates (RTA; UTA; specially trained laypersons to assist in DRE or even serve as a probands for practice) [11,14] and standardized patients [15-17]. Although simulators show high validity [18,19] and lead to reduced inhibition and fear with regard to DRE [20], standardized patients are regarded as one of the most useful methods to deliver DRE skills [16].

Standardized patient (SP) is an umbrella term both for a *simulated patient,* trained to simulate a patient's illness, and an *actual patient,* trained to present their own illness, both in a standardized way [21-23]. SPs are classified as low-technology instruments, which provide a high degree of realism [24] and have strong potential for training general and specific communication [21,25,26] as well as physical examination skills [27-29], with professional feedback seen as the key to their educational success [30-33]. Besides the use of SPs for training general physical examination skills, there is long tradition of using standardized patients for the delivery of intimate examinations, particularly in the area of breast examination skills [34-46] and DRE [15,47-52]. Among the many advantages of deploying SPs is the observation that in their contact with an SP, students are less anxious, particularly within potentially embarrassing examination procedures such as pelvic exams [47].

However, surprisingly little is known about motives, attitudes and initial experiences of SPs who make themselves available to deliver DRE skills. Previous studies have shown that acting as an SP can cause stress and psychological burden [53,54], which is also reflected in psychophysiological measures [55]. Therefore, the aim of the presented pilot study was to learn more about the personal motives and attitudes of SPs as well as their initial training experiences when participating in the DRE for the first time.

## Methods

### Study design

We conducted a descriptive study to investigate the personal background and motivation of SPs who agreed to act as patients upon whom DRE would be performed. We were able to recruit four SPs from the University of Heidelberg’s Standardized Patient Program [56], which enfolds more than 65 SPs in total, to participate in a new training program for delivering DRE skills. All SPs were interviewed after their instructional training session. SPs’ motives, attitudes and training impressions were assessed via semi-structured interviews.

### Standardized patient sample

All SPs (n = 4; 2 female; mean age 48.8 years; for further details see results section) were part of the Standardized Patient Program at the Medical Hospital University of Heidelberg and gave their informed consent prior to their participation in the interview study.

### DRE Training for standardized patients

The aim of the training session for SPs was to qualify them to conduct physical examination skills training sessions for medical students with the topic of examination of the abdomen including pain-free DRE [52]. The training was designed to enable SPs to instruct medical students, to guide role-plays, to adhere to time management, to evaluate the quality of students’ skills performance and to give appropriate professional feedback to students. Prior to the training session, the SPs received a detailed script to be studied in advance. It included information about the program, the role that they would play and the anatomic and technical fundamental principles of the DRE. The SP training was designed in accordance with Peyton’s Four-Step Approach [57], which has been shown to represent a potent method of instruction in previous studies [58,59]. The training encompassed 4 teaching units, amounting to a total of 3 hours. Table 1 shows topics, learning goals and the methodological realization of the SP training session. All SPs were carefully examined by an experienced physician in internal medicine prior to the training sessions and underwent two more examination during the training course.

**Table 1:** **Design of SP DRE training session**

Time	Training steps	Content
Preparation	Introduction	Provision of written script for SPs
Day 1	Overview	● Presentation of goals and focal points of the training program
		● Theoretical overview of anatomy of the abdomen with focus on the rectum in frontal and lateral view
		● Development of the role to be played
	Peyton Step 1	● Video of the examination of the abdomen and DRE
	Peyton Step 2	● Explanation and performance of DRE by the trainer on the SP in a 1:1 setting
	Peyton Step 3	● Recapitulation of examination techniques by SP: One SP performs DRE on a part-task trainer being guided by another SP
	Discussion	● Emphasis on patient safety and discussion of DRE as a “taboo subject” for students
	Peyton Step 4	● Run-through of the complete training scenario. Role-play with SP as doctor
		- history-taking
		- patient education about the examination
		- performance of the DRE on part-task trainer
		- communication of examination results
		● Feedback given by trainer
Day 2	Dress rehearsal	● Performance of the learned examination under supervision of doctor
	First training session	● Field-testing in training session for final year students

### Acquisition of data

Interviews were conducted within a two-week timeframe following the SP training in January 2010 at the University of Heidelberg, Germany on the premises of the Department of Internal and Psychosomatic Medicine at Heidelberg University Hospital. The recruitment of participating SPs took place at the end of DRE training. SPs were informed about the background, goals and course of the study, and participation in the study was voluntary.

### Semi-structured interviews with SPs

This qualitative study examined SPs’ experiences and perceptions of the SP DRE training. The development of the study’s interview questions and hypotheses was undertaken on the basis of an in-depth literature review as well as discussion among a team of experts (N = 5; 2 female, all of whom were experienced in skills-lab and communication training with SPs). We decided against the implementation of group interviews as we wanted to provide a protected environment in which the SPs could talk freely about their personal motives, anxieties or topics that could be marked with shame. The interview manual was constructed in a semi-standardized manner [60-63] and contained the main open-ended questions, followed by encouraging questions and clarifying questions. Main questions addressed SPs’ motives for participating, including the reaction of their social environment, their feelings and expectations before the training and, with respect to their future assignment in students’ classes, their experience of the training and their ideas for improvement (see Appendix for complete interview guideline).

According to the main items of the COREQ checklist [64], in the following, we provide further information about the interview procedure. At the beginning of the interview, questionnaires regarding sociodemographic information and previous work experience were completed by the participants. The individual face-to-face interviews were conducted in person by one of the authors (KD), and were digitally recorded, reviewed and summarized in detailed notes by the interviewer. The interviewer was a female doctoral candidate in her 6th year of medical education training, who had been trained and was supervised by an experienced colleague. The interviews were semi-structured and lasted approximately 15 minutes. The interviewer probed for more details and specific examples when necessary.

### Ethics

The ethics review committee of the University of Heidelberg did not consider this study to require approval. Informed consent was obtained prior to the SP training. We confirm that participation was voluntary, the participants cannot be identified from the material presented and no plausible harm to participating individuals could arise from the study. The study was conducted in accordance with the Declaration of Helsinki (revised form, Seoul 2008). All participants gave written informed consent.

### Data analysis

For the sample description, descriptive statistics were computed (mean, standard deviation). After transcribing the audio files of the 4 interviews verbatim, a qualitative content analysis was performed following the principles of qualitative content analysis and inductive category application [65]. First, we conducted an open coding of all of the 4 interview transcriptions line by line. In detail, single or few sentences were identified as a code, representing the most elemental unit of meaning [66]. Next, the codes were summarized into relevant themes for each participant, using the software MAXQDA (2010 version, VERBI GmbH, Berlin). As themes were recurrent among different participants, they were then compared and adapted until a number of relevant themes for all participants could be defined. The assignment of respective codes to specific themes was conducted by two independent analysers (KD, CN) and subsequently discussed to reach consensus and, if required, adjusted. In the final step, themes were consolidated into three relevant categories.

## Results

### Standardized patient sample

Detailed characteristics of the interviewed SPs are shown in Table 2. The SPs’ occupations at the time of the study were pensioner (SP 1 and SP 3), medical technical assistant (SP 2), and theatre teacher (SP 4).

**Table 2:** **SP characteristics (n = 4)**

SP characteristics	SP 1	SP 2	SP 3	SP 4
Sex [female/male]	Male	Female	Female	Male
Age [years]	54	49	68	24
Previous medical training [yes = 1/no = 2]	2	1	2	2
Years serving as SP [years]	7	5	1	1
Number of roles [n]	12	10	5	1
Physical examination roles [n]	3	4	1	0
Number of attended feedback training sessions [n]	13	8	2	1

### Semi-standardized interviews of standardized patients

With regard to the qualitative analysis of the interview transcripts, all relevant single quotations were identified. From these quotations and codes, eleven themes resulting in three main categories were derived. Main categories were defined as follows: background to program participation (themes A-C); training expectations and experiences (themes D-H); and transfer (themes I-K).

#### Main category “background to program participation” (themes A-C)

##### A) Theme “motives for participating in the training”

When asked about their motives for participating in the DRE training, the SPs indicated that they were mainly there due to intrinsic motivation and interest. One of the SPs was unable to name any concrete motive, but following the training he determined that he had learned a great deal from it. Another SP described his agreement to participate as very spontaneous, without exactly knowing at the time what it would ultimately mean. After receiving further information about the project, however, he stuck to his initial decision.

##### B) Theme “views, expectations, feelings prior to the training”

In response to the question of what views, feelings or expectations the participants had held in the run-up to the training, two of the SPs explained that they had barely given it any advance thought. However, the importance of having the option to withdraw from participation at any time was remarked upon. The prospect of participating in the DRE training was met with curiosity and mixed feelings as well as the expectation of learning something new.

##### C) Theme “talking to others about participation”

Two SPs stated that they had not spoken to anybody from their personal environment about their participation in the DRE training. The main reason given for not speaking about the training was that they feared that others would not understand and they would then have to explain themselves, or else that they didn’t feel the need to talk about it before the training had even taken place. One SP indicated having spoken to his partner about it, but without going into detail. Another SP stated that he had spoken openly about it and that most people had been bemused when they heard that he was participating in this project (Table 3).

**Table 3:** **Main category “background to program participation” (themes A-D)**

Themes	Quotations
**Theme A) Motives for participating in training**	● “The interest, because I enjoy working with students and simply out of interest in medical problems” (SP4)
	● “It just interested me purely from the medical perspective and how one deals with it.” (SP 1)
	● “… I didn’t know exactly what I was letting myself in for and I thought I could imagine myself doing it and because I don’t have any fear of contact in that way and my body can be used for medical purposes so to speak as long as it doesn’t do me any harm, and after I’d assured myself that it is, or could be, a kind of a routine examination I didn’t see any problem with it.” (SP 2)
	● “…I’ve got the time to do it and it’s also very informative for me, I have to say I learned an unbelievable amount.” (SP 3)
**Theme B) Views, expectations, feelings prior to the training**	● “So mixed feelings, curiosity, and I’ll see, if it’s too much for me then I’ll say no, I don’t want to have it done, so in advance I’ve already taken the freedom to say no or I don’t want to do it because it goes beyond my limits or because I don’t want to do it like this.” (SP 4)
	● “It was similar to how I imagined it, I was a bit scared that students would already be examining us today, but otherwise I didn’t give it much thought.” (SP 3)
	● “…in any case I didn’t know what this digital rectal examination area was like and so I was really interested when I flicked through this manual again to see what exactly happens as I have to say, in advance I dealt with it very unknowingly, I knew it was coming but I didn’t know what it is, but then I read through it in advance and I left the option open that if I find it goes too far somehow then I can just go, I mean nobody is forcing me into anything.” (SP 2)
	● “Well, because I knew some of the people involved I trusted that they would arrange it appropriately, so I had a certain trust (…) expectations that I’d learn something from it.” (SP 1)
**Theme C) Talking to others about participation**	● “I think that maybe other people have reservations and you don’t necessarily have to arouse them, I mean, you don’t have to justify yourself.” (SP 3)
	● “Because maybe it would have been indiscrete to talk about something like this (….) that other people would have been rather piqued.” (SP 1)
	● “For some people definitely because of a lack of understanding, because it would be stressful to constantly discuss the same theme with different people or to clarify the same questions which I’ve already answered in my own mind, and I don’t have to justify myself as it were, but rather if it comes to it I can easily talk to anybody about it but it is not that I really want everybody to know that I’m doing something like this or that I’ve got the courage or whatever.” (SP2)
	● “… yes, I wanted to experience it myself before I (…) know whether I want to talk about it.” (SP 2)
	● “Most people looked at me really funny and said you’re letting that be done to you and then I said, sure, it’s interesting and it’s knowledge that you have and I think it’s good to be able to judge, if another doctor does it with me maybe, what it’s like and whether he’s doing everything.” (SP 4)
	● “With my friend (…) but at any rate I didn’t bring it up, so they know that I’m doing this j- doing this this job (…) but I didn’t go into detail.” (SP 2)

#### Main category “training expectations” (themes D-H)

##### D) Theme “preparation for training”

When asked about their personal preparation for the training, the SPs expressed either that they had read the script enclosed in the email sent prior to the training, or that they simply came along to the training.

##### E)Theme “what was important to be able to engage with the training?”

To be able to engage themselves with the training, the SPs found it important to be clear in their own minds that they are ready for such an experience. For one of the SPs, it was important to already know some of the participating team members from previous assignments and to be well prepared. Another SP believed that it would be difficult to find enough training participants and as it wasn’t a problem for him, he signed up for it.

##### F) Theme “embarrassment factor”

Three of the four SPs indicated that they did not have any feelings of embarrassment at any time during the training. They believed that this was primarily due to the professional implementation and pleasant atmosphere during the training or else they attributed it to their personal biographical experiences, which had led to the fact that they did not experience such a situation of exposure as embarrassing. One SP found the DRE carried out on him by a lecturer during the training to be embarrassing, which he believed was mainly down to the fact that it was a very unfamiliar situation for him. Nevertheless, he also found that the training personnel did everything they could to limit the embarrassment. The SPs found that the lecturers dealt with the potentially embarrassing theme in a very empathetic and appropriate way.

##### G) Theme “how the training was experienced”

The SPs responded unanimously that they experienced the training very positively, as interesting, informative and empathetic. However, in part, the issue was raised that participation in the training is not an everyday activity and that it was a physically very demanding experience. When asked whether he had found anything to be stressful or unpleasant during the training, one SP responded that he had some doubts as to whether he could reconcile himself with the idea of earning money from the participation in the DRE training. The other SPs stated that they did not find anything to be stressful or unpleasant.

##### H) Theme “suggestions for change”

In response to the question whether anything should be changed about the training, the SPs responded unanimously that they would not change anything about the general concept. In part, however, it was pointed out that following the application in practice, other suggestions might emerge. Following more targeted questioning, it was mentioned on several occasions that in part, the SPs would have wished for more repetitions and implementations of the DRE. Otherwise, the SPs were of the opinion that it would be good to have been informed of clear action guidelines in case conflicts with the students emerged in the later teaching, and that a compact summary regarding the training contents which they could take away with them would be helpful. Moreover, it was mentioned that there should be clearly defined breaks and that it would be nice to be offered coffee or such like. On one occasion it was mentioned that at times there were too many lecturers present during the DRE training (Table 4).

**Table 4:** **Main category “training expectations” (themes D-H)**

Themes	Quotations
**Theme D) Preparation for training**	● “I read the manual and learned the role of Althoff” (SP 1)
	● “I just came along.” (SP 3)
	● “By the fact that I got the script (…) and then also looked there.” (SP 4)
**Theme E) What was important to be able to engage with the training**	● “(…) that I knew at least some of the people involved personally (…) that I was well prepared.” (SP 1)
	● “… but then I though that in some place a progressive approach is also useful for medicine (…) and otherwise I don’t really have much fear of physical contact, although it was completely new to me I just chalked it up as a physical experience for me.” (SP 2)
	● “(…) and I thought again about how I would feel when I’m being examined like this, what is it like for me when somebody gets too close to me or goes beyond my embarrassment threshold at that moment, but then I found it OK and I found it very interesting to experience it myself in this way (…) that someone does it on me and I also evaluate it.” (SP 4)
	● “For me, it is an examination almost like any other, this is also a reason why I said yes, and I was of the opinion that they wouldn’t find many people, and that was the case, and I thought I don’t mind it, I’m happy to take part.” (SP 3)
**Theme F) Embarrassment factor**	● “Because the conditions were pleasant, I think that I might have perceived the same training but with different personnel differently, but in this constellation how it was with us I found it very good, there were always enough people there to support you so in each sub-group there was always erm at least one person present who guided it, yes.” (SP 2)
	● “Because I found that it was presented very carefully and very naturally, it was not evaluated as very embarrassing by those who were presenting it, so I found that very good (…) it was just good, it was presented as something really natural and I found it easy to engage myself with it.” (SP 4)
	● “There weren’t any moments when I felt naked as it were, because there was always either a piece of clothing laid over you or there was a cover, and the doctor really avoided making total eye contact or whatever and this = this feeling of standing there naked in front of someone who was dressed or whatever (…).” (SP 2)
	● “So this rectal examination, it was a bit embarrassing, but they really did everything they could to limit the embarrassment (…) I didn’t know how I would react to this rectal examination, so for that reason.” (SP 1)
	● “Yes, appropriate, so they addressed it really well.” (SP 1)
	● “So I found it very empathetic today (…) it was simply good.” (SP 3)
	● “So very good, as very sensitive.” (SP 4)
	● “It was dealt with really OK, it was very clear (…) so I didn’t have any doubts in the personnel at any point.” (SP 2)
**Theme G) How SPs experienced the training**	● “Very pleasant, very gentle, discrete and sensitive.” (SP 1)
	● “(…) very relaxed, it was good (…) the session was kind of doable in terms of time, it was pleasant, there were not too many people there (…) the training today was kind of the most intensive, so from a purely physical point of view because it was just a completely new type of examination for me and because I didn’t have any experience with it, so also unpleasant, but also very interesting kind of for that reason.” (SP 2)
	● “So I found it very good, very informative for me and I think it will all stick.” (SP 3)
	● “I experienced it as very interesting and also enriching because there were a lot of things I didn’t know.” (SP 4)
	● “Whether I can, as it were, reconcile in my own mind that I am earning money for it or on the one hand it is of medical use and on the other hand I’m getting money for it and then I didn’t really know how to weigh that up in my mind, and that’s the moment when I had my doubts.” (SP 2) for it or on the one hand it is of medical use and on the other hand I’m getting money for it and then I didn’t really know how to weigh that up in my mind, and that’s the moment when I had my doubts.” (SP 2)
**H) Suggestions for change**	● “Was actually good how it was, yes, I wouldn’t change anything about it.” (SP 1)
	● “So, I found again today that it was very illustrative, so with all possible means as it were, so with videos and with a model and through through a presentation and then using practical so the practical examination situation, and it was very very rounded and very diverse, yes, so.” (SP 2)
	● “So, I felt really at ease, you could, well purely from a technical perspective I have the feeling I am well informed (…) the documents, the materials are also clear.” (SP 2)
	● “Well, for the moment I wouldn’t change anything about the concept, we’ll see how the whole thing can be applied in practice (…) and then maybe some conclusions can be drawn” (SP 2)
	● “Erm, well, the lecturers have in principle done a very good job, they dealt with things very well, so I haven’t got anything at all to add there.” (SP 2)
	● “And different lecturers, which also constantly refreshes your attention if you’re not listening to the same person for four hours, I think that’s also good.” (SP 2)
	● “Er, I think this whole, erm thick manual, it could have been a bit thinner, today she gave us a summary now about the feedback, I think that’s really good.” (SP 3)
	● “So, I wouldn’t pack any more into it, so if you’ve got it in this framework (…) so I would I would rely on repetition more, so no I don’t mean the repetition of the exam-, so if at all then I would rely on on repetition, so that you maybe just do it twice so that it can all sit better.” (SP 2)
	● “Why the first feedback didn’t really work because I had the feeling that the feedback (…) was repeatedly addressed and brought up (…) but ultimately, the only thing that really matters (…) that you get it across and you’ve got all these aids how you can implement the different treatment steps that you really practice it more frequently, so you don’t just talk about it, which is also important but that you actually do it more often (…) or you watch others doing it more often.” (SP 2)
	● “Hmmm, err, I, the maybe a fourth session so not cut short (…) rather rather errm train (…) the respective treatment step in practice a bit more extensively.” (SP 2)
	● “When there are conflicts er between the students and and SPs, yes in that case the SP has to in effect go some way to replacing the lecturer and dealing with conflicts in the situation (…) so that I at least, I should say, that we are errm, told how to deal with it, yes so if for example I erm might have to give feedback to medikit employees er or whether I don’t have to give feedback, I don’t want to stand there as an in-informer, do I?” (SP 1)
	● “Yes, what I just always wish for is a precisely defined break, so that y-you know what you can get done in the break and what you can’t.” (SP 1)

#### Main category “transfer” (themes I-K)

##### I) Theme “advantages of this teaching method from the SPs’ perspective”

According to the SPs, the advantages of participating in the DRE training and the DRE program were that the students received feedback from the patients’ perspective, which might lead to the students feeling more secure in implementing DRE. One SP responded that he didn’t feel confident to judge this because he didn’t precisely know how a DRE was previously taught, but that it must be good to gain practical experience and to be given feedback from the patient perspective.

##### J) Theme “How SPs view the prospect of the assignment with students”

The majority of the SPs answered that they had no concerns regarding their assignment in the teaching and that they felt well prepared following the DRE training. One SP responded that he was not currently feeling very relaxed, but that this would no longer be very relevant when he had looked through the given role play instructions at his leisure at home before the DRE teaching. One SP indicated being slightly afraid of forgetting something. The SPs were also asked whether they had any misgivings regarding their assignment in the student teaching. Mostly, no fears could be mentioned. However, in isolated cases there was a fear that the students would not be able to respect the SPs’ privacy or that no authentic examination situation would arise.

##### K) Theme “what is particularly important for application in student teaching?”

In response to the question of what, based on their current experience, is particularly important for the implementation of the DRE in the student teaching, the SPs stated that good preparation, respect for the sense of embarrassment, good communication and a positive working atmosphere were important. One SP also hoped that the students would learn something from the teaching and then be in the position to transfer what they had learned to practice (Table 5).

**Table 5:** **Main category “transfer” (theme I-K)**

Themes	Quotations
**Theme I) Advantages of this teaching method from the SPs’ perspective**	● “Directly, so to speak, after the examination he is told directly what he did wrong or (…) he gets the feelings as a patient reflected back to him.” (SP 1)
	● “I can’t exactly judge it because I don’t know how it was previously, what was lacking there as it were, or what gaps there were, so I think that, as it were, if these teaching units didn’t take place in the past, or were less intensive, then it leads in the students’ training that one guarantees giving the students the possibility to do a lot of practical work and beyond this (…) directly from the patients’ perception, which is of course somehow very subjective and maybe not as technically competent as that of a doctor or patient, but also precisely for this reason so interesting, because it is so diverse.” (SP 2)
	● “I now imagine myself in the in the role of a student and think then that it is considerably easier than if I go directly to a patient (…) maybe it takes away a certain insecurity (…) they become more secure through it, because trying it out on a patient is certainly more anxiety-ridden than on a student.” (SP 3)
	● “The fact that the students can practice how it is with a patient and now deal with it in such a careful and empathetic way (…) so not just theory but that there is also really practice behind it.” (SP 4)
**Theme J) How SPs’ view the prospect of the assignment with students**	● “So I didn’t have any concerns whether it will work (…) and yes, provided the student plays along.” (SP 1)
	● “I think, like the other assignments, that we’re actually well prepared, so that we can get started now.” (SP 2)
	● “I’ll look at it again in peace and quiet at home, I have to reflect on it again, it has to sit, I realise that it is not yet all at ease, not so relaxed but I think when I’ve read it again and trained it again then it’ll be completely OK.” (SP 4)
	● “So I’ve still got a bit of a problem with the feedback, I’m just a bit scared that I’ll forget something, so that means I have to always look at everything again before the next assignments.” (SP 3)
	● “Maybe that the student will not deal with the situation appropriately, so for example, doesn’t pay much attention to my sense of embarrassment or something.” (SP 1)
	● “The fact that this patient-student dialogue is not authentic, that there’ll somehow be something acted there, which definitely depends a lot on the SPs but not only on them (…) because the student also plays a large role and that is also an opportunity for the student, what he ultimately draws from it is up to him, but there’s the fear that people will slip out of their roles (…) that no authentic treatment provider situation will come about.” (SP 2)
	● “There aren’t any fears, at the moment I can’t think of anything.” (SP 4)
**Theme K) What is particularly important for the application in student teaching?**	● “Being thoroughly prepared (…) that you are certain about the examination and the individual examination steps and about the possibilities of making mistakes.” (SP 1)
	● “That the communication goes well that the different treatment steps are adhered to and that a pleasant working atmosphere arises and that it is checked whether the student is in a position to do it and therefore is also on the right path in his development to become a doctor.” (SP 2)
	● “Communication with an uncomplicated patient and just that they can try it out (…) and also I know how it should work, the whole thing, the examination, after all, a normal patient can’t give this feedback like I can.” (SP 3)
	● “Respecting the patient’s embarrassment threshold and communicating with him during the examination.” (SP 4)

## Discussion

The presented study elucidates personal motives of SPs for participating in intimate physical examination training for the subsequent delivery of digital rectal examination (DRE) skills. Moreover, it examines impressions regarding an experienced training session as well as expectations related to the first DRE teaching session with medical students. The results show that there are no obviously predominant motives for DRE program participation. SPs participate in the DRE training sessions with relatively little prejudice and anticipate no distinct vulnerability within teaching sessions with undergraduate medical students.

Surprisingly to us, there do not seem to be any predominant personal motives, e.g. altruism, for SPs to participate in the DRE program. SPs participate without prejudice, without anticipating risks or burden, and mainly without broaching a controversial issue within their own social environment. Accordingly, participation is mainly *not* discussed with friends or relatives, although if it is discussed, the social environment reacts with bemusement. On the one hand, this laid-back behaviour of the SPs could be interpreted as sign of the professionalism and in-depth experience of the SPs examined in our study [56]. On the other hand, however, our findings show that detailed educational advertising on the program and the training is needed, which addresses potential personal psychological reactions before, during or after participation in DRE training or teaching sessions, as well as possible reactions of bemusement within the social environment. Indeed, adolescent standardized patients portraying adolescent roles reported discomfort but no long-term adverse effects of participation, especially when questioned about their sexual history [67]. In a qualitative focus group study, all of the 16 examined SPs described psychophysiological effects when portraying emotionally intense roles, sometimes lasting for several days [54]. Recent literature revealed that SPs show psychophysiological reactions in terms of a diminished heart rate variability during history-taking encounters, indicating emotional stress [55]. These psychophysiological reactions may even be much more pronounced when delivering intimate physical examination skills.

Within our SP program, SP selection processes encompass comprehensive information talks [56]. Hanson et al. [67] proposed a two-component SP selection consisting of an employment component (30-minute interview on work history, attitudes towards the medical profession, and health and background variables that might affect SP participation and performance) and a psychological component (psychological questionnaire assessment). An all-embracing information talk that incorporates potential side effects is an ethical imperative, as the ethically awkward aspect of SP performances is that although they are intended to protect “actual patients” from risk and suffering, they cannot avoid imposing a certain degree of risk and suffering on other people: the SPs themselves [68].

Regarding the expectations towards the training session with undergraduate students, again, the SPs’ comments reflect similar attitudes. SPs do *not* worry about what they will be confronted with during the training sessions. However, they do stress the importance of being allowed to withdraw from training participation at any time during the training, revealing more deep-seated worries that are merely touched upon by SPs. In line with this, SPs wish for the training to be conducted by a team of experts with whom they are familiar. The aspect of the possibility to withdraw from training participation should be actively addressed in preceding SP briefings and if possible, the desired familiarity within the training session, with team members who are already known to the SPs, should be realized. These offers could serve to reduce anxiety and worries, which SPs seem to find difficult to address, and should be an integral part of the DRE training.

The DRE training itself was not experienced as being embarrassing. The atmosphere was perceived to be professional, appropriate and comfortable. Only one of the SPs felt a sense of embarrassment, although none of the SPs experienced training aspects as being displeasing or stressful. Furthermore, the training session was regarded as interesting, informative and empathetic. This indicates that the proposed training model incorporating a design oriented to Peyton’s Four-Step Approach [57] could act as a model for the training of SPs serving for physical examination or intimate examination skills in general. A similar training model was proposed for SPs willing to teach physical examination skills who were trained by physical examination teaching associates in a 3 h-session for each organ system, encompassing video demonstration, training on each other, and finally the case being taken over by medical doctors [69]. However, the presented training model is the only model to be published and proposed for the training of physical and intimate examination skills including step 3 of Peyton’s Four-Step Approach that has been shown to be efficient in the acquisition of clinical skills [58]. Nevertheless, although the training concept was well received, there was still a wish for the opportunity for deliberate practice. This is an important advice from the SPs, as deliberate practice is indeed one of the most relevant factors for the successful acquisition of skills learning [70]. Furthermore, SPs suggested compiling guidelines to handle difficult situations in DRE training with participating students, as has been proposed and well received in other fields of medical education [26].

When asked about the advantages they experienced from using the SP method to deliver DRE skills, the interviewed SPs stressed the possibility for students to actively train DRE on real human beings and to receive feedback from the patient perspective. Indeed, the active training and supervision of DRE skills during medical education is rare [4-10]. Furthermore, final year students complain about a lack of supervision representing the most relevant hindrance for the acquisition of DRE skills [7]. In this respect, supervised student training and constructive feedback is urgently needed, as feedback represents one of the most effective methods for behaviour modification [71]. In terms of their expectations regarding their first assignment in curricular medical education training, SPs feel well prepared, but – although they are very experienced – they fear that their privacy could be invaded, which could lead to intrapsychic stress and prevent them from creating an authentic atmosphere. They wish for their private sphere to be respected and for the establishment of a reliable working atmosphere. Therefore, instructive advice for students and information for SPs on this matter could be an important factor for reducing anxiety and achieving a fruitful learning environment.

In summary, the following guidelines for recruitment, SP training and preparation for teaching sessions can be derived from the SP interviews conducted:

- Exclusively appointing experienced SPs (previous experience in delivering physical examination skills)
- Conversation about the SPs’ motives for participating in the program, addressing possible worries and fears
- Clarifying that participation in a DRE program can lead to mental strain in the SPs and bemusement from the social environment
- Information about whom the SPs can turn to if they experience subjective stress
- Detailed information about the course of the SP training with the goal of achieving a reduction in anxiety
- Actively addressing the possibility to withdraw from the training at any time
- Creating calm, protected professional conditions within the SP training
- Presence of and support by the team members, with whom the SPs are already familiar
- Development and handing out of action guidelines for dealing with students who behave inappropriately
- Information given to students about ensuring respectful conditions during the teaching events
- First teaching assignments only in the presence of and supported by the personnel who are familiar to the SPs

### Limitations

Several limitations of the current study have to be mentioned. First, the sample size was rather small, although we were able to include all of our SPs who are part of the DRE program. This potentially limits the representativeness of the study and possibly results in the themes within the qualitative analyses not being exhaustive. Furthermore, due to the exploratory nature of this research, the generalisability of our findings may be restricted. However, to our knowledge, the presented study is the first to assess motives, experiences and expectations of SPs in a DRE program in a qualitative, in-depth analysis.

## Conclusions

In conclusion, the current study examined SPs’ motives, views, expectations and experiences regarding a DRE program during their first training experiences. The results enabled us to derive distinct action guidelines for the recruitment, informing and briefing of SPs who are willing to participate in a DRE program. Further research should address long-term distress related to program participation, differential perceptions of different training settings, and further qualitative research on SPs’ teaching experiences.

## Appendix: interview guideline

The interviewer is asked to read the questions exactly as written, followed by encouraging and clarifying questions:

1. How did you experience the training course?
  1.1 Did you experience individual elements of the training course as shameful?
  1.1.1 What exactly did you experience as shameful?
  1.1.2 What was not shameful for you?
  1.1.3 Why were these training elements not shameful for you?
  1.2 What was important for you to be able to engage in the DRE training course?
2. How did you experience the lecturers dealing with the shameful issue?
3. What was stressful or unpleasant for you during training?
4. You participated in the training for Standardized Patients for the simulation of the digital rectal examination. What made you decide to participate in this training course?
5. Have you spoken to your relatives/acquaintances about your participation in the project?
6. What experiences did you make?
7. What were your motives for not talking about your participation in the training/training in the project?
8. What ideas, expectations and feelings did you have prior to the digital rectal examination training course?
9. How did you prepare yourself for the training course?
10. What would you change in the concept of the training course?
11. Is there something that you would change about the conditions of the training course?
12. Is there something that you would change about the sequence?
13. Is there something that you would change in the interaction with the instructors?
14. You have been trained to give students feedback on the digital rectal examination. What are your feelings in light of this assignment?
15. What is beneficial about the use of this examination method in teaching in your view?
16. What concerns are there for the use of this examination method in undergraduate teaching in your view?
17. What do you consider to be particularly important for use in undergraduate teaching in light of your current experience?
18. Has something been left unmentioned that you think is important?
